# Serum and Tissue HIF-2 Alpha Expression in CIN, *N*-Acetyl Cysteine, and Sildenafil-Treated Rat Models: An Experimental Study

**DOI:** 10.3390/medicina54040054

**Published:** 2018-07-30

**Authors:** Ismail Altintop, Mehmet Tatli, Cigdem Karakukcu, Zeynep Soyer Sarica, Arzu Hanım Yay, Esra Balcioglu, Ahmet Ozturk

**Affiliations:** 1 Department of Emergency Medicine, Kayseri Training and Research Hospital, 38010 Kayseri, Turkey; drmehmettatli@gmail.com; 2 Department of Biochemistry, Kayseri Training and Research Hospital, 38010 Kayseri, Turkey; ckarakukcu@hotmail.com; 3 Experimantal Research Center, Erciyes University, 38010 Kayseri, Turkey; zeynepsoyer_94@hotmail.com; 4 Department of Histology and Embryology, University of Erciyes, 38010 Kayseri, Turkey; arzu.yay38@gmail.com (A.H.Y.); esrabalcioglu79@hotmail.com (E.B.); 5 Department of Biostatistics, Erciyes University Faculty of Medicine, 38010 Kayseri, Turkey; ahmets67@hotmail.com

**Keywords:** contrast-induced nephropathy, hypoxia-inducible factor 2α, *N*-acetyl-cysteine, sildenafil

## Abstract

*Background and Objectives*: Contrast-induced nephropathy (CIN), is acute renal damage due to contrast agents. This study is conducted to evaluate serum and renal heterodimeric nuclear transcription factor (HIF)-2 alpha levels and its tissue expression in contrast-induced nephropathy, and in *N*-acetyl cysteine (NAC)-and Sildenafil-treated rat models. *Materials/Methods*: This randomized, controlled, interventional animal study was conducted on Wistar rats. Rats (*n* = 36) were randomly assigned to four groups: control (*n* = 9), CIN group (*n* = 9), CIN *+* NAC group (*n* = 9), and sildenafil (*n* = 9). The rat model was used to form iohexol-originated CIN. During the modeling, prophylactic treatment was performed at the 24th and 48th h. After 48 h of modeling, blood, urine, and tissue samples were obtained for biochemical analyses. HIF-2-α levels were measured in renal tissue, serum, and urine samples. Renal sections were also performed for histopathologic and immunohistochemical evaluations of renal injury and HIF-2-α expression. *Results*: In the CIN model, HIF-2α levels and other biochemical parameters were significantly increased (*p* < 0.01). Both sildenafil and NAC efficiently decreased renal damage due to contrast agents, as shown in histopathologic examinations (*p* < 0.05). Similarly, after treatment with sildenafil and NAC, HIF-2α levels were significantly decreased (*p* < 0.05). *Conclusions*: The current study shows that serum and tissue HIF-2α levels decrease in CIN. Besides, the levels and tissue expression of HIF-2α decrease with both NAC and sildenafil treatments. With further studies, HIF-2α can be investigated as a biomarker of CIN and can be used in the follow-up of patients with CIN.

## 1. Introduction

Contrast-induced nephropathy (CIN) is a form of acute renal damage resulting from the use of contrast agents. Diagnosis of CIN is made with 25% increments or 5 mg/dL increases of serum creatinine (sCr) versus basal levels within 48 h of contrast agent usage [1]. In recent years, along with rapid developments in medical imaging techniques, examinations and treatments with intravenous contrast agents in emergency services or other services may induce CIN. CIN is one of the most important causes of acute renal damage/injury in patients treated at emergency services and in inpatients [2,3,4]. In the current literature, the rates for CIN are reported to be between 0.2–2.0%, after tomography was undertaken with contrast agents [1].

Pathophysiologically, CIN is closely associated with renal hemodynamic changes, medullar ischemic injury, oxidative stress injury formed with reactive oxygen species (ROS), secondary damage to tubules, and tubular obstruction [5]. In many experimental studies, chronic hypoxic damage is pointed out as an eventual common cause for the progression of chronic renal disease (CRD) to end-stage renal failure [2]. Thus, therapeutic interventions for hypoxia may be a valid tool to cease CRD.

Hypoxia-inducible factor (HIF), a heterodimeric nuclear transcription factor, is a crucial intermediate form for protection mechanisms against hypoxia. HIF forms reactions to preserve renal hypoxic tissues and to decrease the damage after the decrease in hypoxia [6]. In chronic and acute renal failure, HIF is being activated [7,8]. There are studies emphasizing HIF activation in chronic renal fibrosis in CRD [6]. In situations like CIN, acute hypoxic renal damage occurs [2,5]. The decrement in intramedullary blood flow is secondary to hypoxia and direct tubular damage induces CIN [2,9].

There are two well-known forms of HIFα: HIF1α and HIF2α [6]. In other studies, HIF2α is detected to be higher in renal cells and is responsible for erythropoietin production [10,11]. In the literature, HIF2α levels were demonstrated to be specific to renal cells [7]. The increment in HIF2α levels under hypoxic conditions is a key mediator for cellular oxygen homeostasis [12]. In a number of experimental studies conducted with unstable metals like cobalt and nickel, hypoxia-induced an increment in HIFs and had a renal-protective effect [13]. Accordingly, HIF plays an important role in acute renal injury and is the most important factor in the development of hypoxia, inflammation, and angiogenesis [7]. Pinelopi et al. [14], detected HIF2α levels to prevent ischemic renal injury.

In a vast number of studies, risk factors and prophylaxis strategies for CIN have been determined. Except for volume therapies, there is no consensus or an exact protocol for the use of these strategies in emergency services. *N*-acetyl cysteine (NAC) is commonly used for the treatment of CIN [1]. Also, in recent years sildenafil has been determined to be effective in experimental CIN models [15].

Nephropathy scoring is used in order to test the efficacy of contrast nephropathy treatments [1]. There is no current biomarker being used for diagnosis and monitoring of CIN. Normal blood urea nitrogen (BUN) and creatinine levels do not point out the absence of CIN. In the current literature, there is no specific biomarker that has been identified to demonstrate CIN. Therefore, in the current study, we aimed to evaluate serum and renal HIF-2α levels and tissue expression of contrast-induced nephropathy, and *N*-acetyl cysteine (NAC)-and sildenafil-treated rat models.

## 2. Material and Methods

### 2.1. Experimental Materials

All procedures using animals in this study were approved by the Ethical Committee of Erciyes University Experimental Research and Application Center (Approval date and number: 14 June 2017 17/063). Thirty-six, 16-week-old Wistar albino female rats weighing 200–250 g in the same condition were selected (Erciyes University Experimental Research Center). They were provided with adequate commercial feed (produced by Purina, Düzce, Turkey) and tap water. The rats were arranged into four groups, and each group was arranged in four cages (25 × 40 × 20). Each cage contained two or three rats, and coarse sawdust bedding (Kayseri, Turkey). Rats were accommodated under conventional experimental animal housing conditions with controlled temperature (23 ± 2 °C), humidity (50 ± 5%), and air change (12 air change per hour), 12 h of light and darkness, and ad libitum feed. The general health status of the rats was monitored prior, during, and at the end of the study.

NAC was purchased from Basel Pharmaceutical Co., Ltd. (Istanbul, Turkey), sildenafil was purchased from Actavis Pharmaceutical Co., Ltd. (Istanbul, Turkey), and low-osmolar, non-ionic contrast media agent (Iohexol) was obtained from Opakim Pharmaceutical Co., Ltd. (Istanbul, Turkey).

### 2.2. Model and Grouping

**Figure 1:**
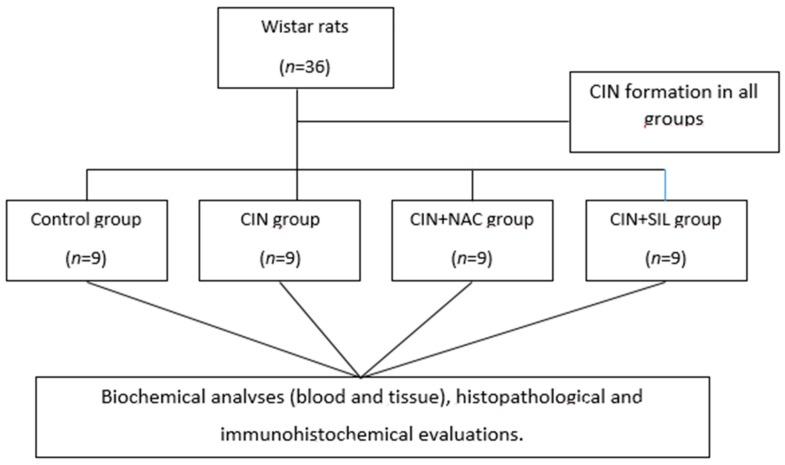
Flow chart of the study. (Contrast-induced nephropathy (CIN), Contrast-induced nephropathy *N*-acetyl cysteine (CIN + NAC), Contrast-induced nephropathy Sildenafil (CIN + SIL)).

The rats were randomly assigned to four groups: (a) control group, (b) CIN group, (c) CIN plus NAC group, and (d) CIN plus sildenafil group, with nine rats in each group (Figure 1). CIN rats were subjected to CIN protocol as follows: [16,17]. Rats in the CIN model, NAC, and in the sildenafil group were anesthetized with 60 mg/kg pentobarbital. Pentobarbital sodium anesthesia was followed by CIN induction, which was performed with drug administration into a tail vein. Drugs administered consisted of low-osmolar, non-ionic contrast medium agent (Iohexol) at a dose of 1600 mg iodine/kg. This is the standard contrast medium dose for clinical purposes and other related experiments in rat studies [5,17,18]. For each time period, control group rats were injected with equivalent amounts of saline, in terms of volume. Rats in the NAC group received intragastric administration of NAC (150 mg/kg) 48 h prior to the CIN-inducing injections. Rats in the sildenafil group also received intragastric administration of sildenafil (50 mg/kg) 48 h prior to the CIN-inducing injections. The control group and the CIN group were given an equal volume of saline by intragastric administration.

After the injection protocol, rats in all groups were put into their routine nutritional environment. In addition to the contrast agent-provided hours, with a minimum of 48 h and under anesthetic conditions, blood and tissue samples were obtained from rats, and the blood and serum markers were measured. A total of 5 mL of intracardiac blood samples were taken from rats under ketamine/xylasine anesthetics. Control groups and experimental groups were sacrificed concurrently. After being dispensed into dry tubes, blood samples were centrifuged at 3000 rpm for 10 min. The obtained serum samples were stored at −80 °C until analysis.

### 2.3. Biochemical Analyses

Serum, urine, and tissue HIF-2a levels were detected by a commercial kit using the quantitative sandwich enzyme immunoassay technique (Human HIF2a ELISA kit; SunRed Biotechnology Company, Shangai, China). Serum creatinine was measured with a modified Jaffe’s reaction, and urea was measured by coupled enzymatic method by an Autoanalyzer (Beckman Coulter AU 5800, Atlanta, GA, USA).

### 2.4. Biochemical Evaluation of Tissue Samples

Renal tissue samples of rats were cut in the middle, and weights were adjusted to 0.25 g. Frozen tissues and 1 mL of phosphate-buffered saline (pH 7.4) were placed in a 2.0 mL screw cap tube with 0.4 g sterile zirconium beads (0.3 g of 0.1 mm and 0.1 g of 0.5 mm). Tubes were placed in the BeadBug™ (D2400 BeadBlaster 24 Microtube Homogenizer, Edison, NJ, USA) and processed for 1 min and six cycles with 30 s intervals, at 6.5 m/s. Tubes were incubated in a cold nitrogen tank for 3 min, and the same process was repeated in the homogenizer. Tubes were centrifuged at 4 °C, 16,000× *g* for 10 min. Supernatants were transferred to a fresh 2.0 mL tube for further analysis.

### 2.5. Histopathological Evaluation

For histological examination, routine paraffin wax embedding procedures were used. The kidneys were removed, divided into sections, fixed in 10% formalin, and processed by routine histological methods. After embedding in paraffin, 5 μm thick paraffin sections were removed from each sample and placed on poly-l-lysine slides. In order to evaluate the morphological characteristics of the tissue and structure before assessment by light microscopy, all sections were colored with hematoxylin-eosin (H&E) (Olympus BX51, Tokyo, Japan). Renal injury was graded as follows: At least 10 random, non-overlapping fields (200× magnification) were observed for each slice, and afterwards, the mean percentage of the injured renal tubules was calculated. The following grading system was implemented for the histopathological evaluation of tissues under light microscopy: No damage was marked as 0; <25% damage was marked as 1; 25–50% damage was marked as 2; 50–75% damage was marked as 3; >75% damage was marked as 4 [19].

### 2.6. Immunohistochemistry

The renal tissues were fixed in 10% buffered formalin solution and after routine laboratory methods, were embedded in paraffin. Five µm-thick paraffin tissue sections were placed on poly-l-lysine slides. The slides were air-dried, and the tissue was deparaffinized. Tissue sections 5 µm thick were rinsed in de-ionized water, antigen retrieval was performed by incubation in 10% citrate buffer (pH 6.0) at 300 W for 10 min, and sections were afterwards cooled to room temperature for 20 min. The sections were incubated in 3% H_2_O_2_ for 10 min, then rinsed in phosphate-buffered saline (PBS). An Anti-Polyvalent HRP DAB detection system kit (Thermo Scientific, Waltham, MA, USA) was used for the following steps. To reduce non-specific staining, sections were pretreated with normal block serum for 20 min. Primary antibodies used were raised against HIF2α (HIF-2 alpha polyclonal antibody, cat no PA1-16510). The slides were incubated overnight at 4 °C in a humidified chamber. After washing three times for five minutes in PBS, sections were incubated with biotinylated secondary antibodies for 15 min. After washing in PBS, 3,3 P-diaminobenzidine tetrahydrochloride (DAB) was applied as a chromogen, and the sections were counterstained with hematoxylin. The stained sections were examined for HIF2α immunoreactivity under an Olympus BX-51 light microscope (Olympus BX-51, Tokyo, Japan). Two histologists continuously observed at least 10 high-power fields (200×) for each slice and calculated the immunoreactivity intensity to reflect the intensity by using ImageJ software, Bethesda, MD, USA [20].

### 2.7. Quantitative Immunohistochemistry

Quantitative immunohistochemistry and histomorphometry were performed using ImageJ software. The Terminal deoxynucleotidyl transferase dUTP nick end labeling (TUNEL)-positive cells were counted in the kidney tissue sections without distinguishing the cortex and medulla. Immunoreactivity intensity values for HIF2α were calculated for sections in which HIF2α staining was applied.

### 2.8. Statistical Analyses

Statistical analyses were performed by SPSS 22.0 (Chicago, IL, USA). The normality of the data was assessed by the Shapiro-Wilk normality test and Q-Q graphs. One-way analysis of variance (ANOVA) (post hoc test) was used to compare BUN, sCr, Urine urea, Urine Cr, HIF-2α-tissue, HIF-2α-serum, HIF-2α-urine, and QIRIAR (Quantitative immunohistochemistry score) results. The Kruskal−Wallis (posthoc Dunn’s and Benferroni) test was used to compare tubular damage score in groups. Statistical significance was set at *p* < 0.05.

## 3. Results

There was no death among the rats in this study. There were no significant anomalies in the nutrition or activity of rats in groups. A CIN model was formed, and the parameters were measured in the CIN model. Renal functions, HIF-2α levels, and QIRIAR were compared among groups (Control, CIN, CIN *+* NAC, CIN *+* HIF) in Table 1.

### 3.1. Comparison of Renal Function Among the Four Groups

When renal function variables were compared between groups, BUN and sCr were detected as significant (*p* < 0.001), while urine BUN and urine Cr variables were non-significant (*p* = 0.678 and *p* = 0.788, respectively). According to the multiple comparison test (post-hoc test: Tukey), BUN was significantly different in CIN *+* SIL and CIN groups (*p* < 0.05) versus the control group. According to the same test, sCr was not significant between the second and third groups (*p* > 0.05). Other possible pairwise comparisons were statistically significant (*p* < 0.05) (Table 1).

### 3.2. Comparison of HIF-2α Levels Among the Four Groups

HIF-2α-serum, HIF-2α-tissue, and HIF-2α-urine values were measured in the control group, and the effects of SIL and NAC on CIN rats were shown in Table 1. When SIL and NAC were given to rats in the CIN group versus the CIN group, serum HIF-2α levels and kidney tissue HIF-2α levels were both decreased. As the HIF-2α levels were compared between the different groups, kidney tissue (ng/g) and serum levels were significant (*p* < 0.001); however, urine levels were non-significant (*p* = 0.382).

According to multiple comparison tests (post-hoc test: Tukey), tissue HIF-2α levels were significant for the CIN group and the control group, CIN *+* SIL, and CIN *+* NAC groups. Additionally, the difference between the control and CIN *+* NAC groups was also significant (*p* < 0.05) (Table 1).

Differences between groups in terms of QIRIAR numbers were significant (*p* < 0.001). According to the multiple comparison test (post-hoc test: Tukey), all possible dual comparisons were significant (*p* < 0.05) (Table 1).

According to the multiple comparison test (post-hoc test: Tukey), BUN was significantly different in CIN *+* SIL and CIN groups (*p* < 0.05) versus the control group. Other possible pairwise comparisons were statistically significant (*p* < 0.05).

**Figure 2:**
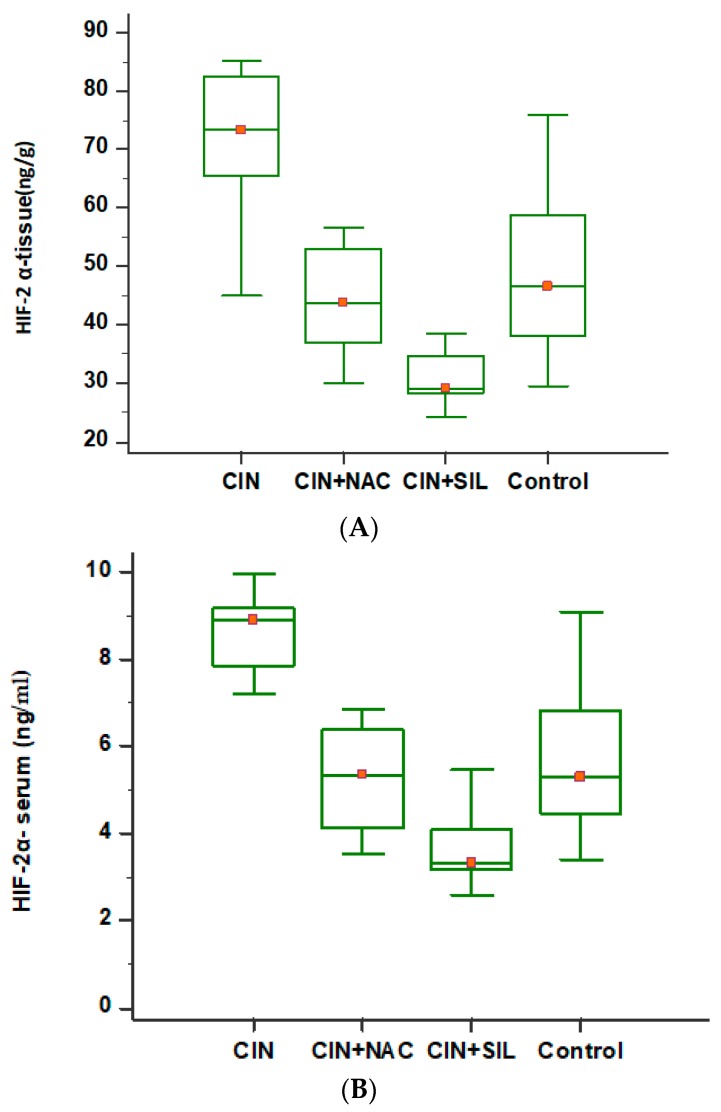
Multiple comparison test of HIF-2α-tissue (**A**) and HIF-2α-serum (**B**) for all groups. According to multiple comparison tests (post-hoc test: Tukey), tissue HIF-2α levels were significant for the CIN group and the control group, and CIN *+* SIL and CIN *+* NAC groups. Additionally, the difference between the control and CIN *+* NAC groups were also significant (*p* < 0.05).

In Figure 2, comparative serum and tissue HIF-2α levels are given for all groups, as shown with box-plot graphics.

### 3.3. Effects of Sildenafil on Kidney Histopathological Alterations, Histopathologic Findings in CIN Rats and Treatment Groups

**Figure 3:**
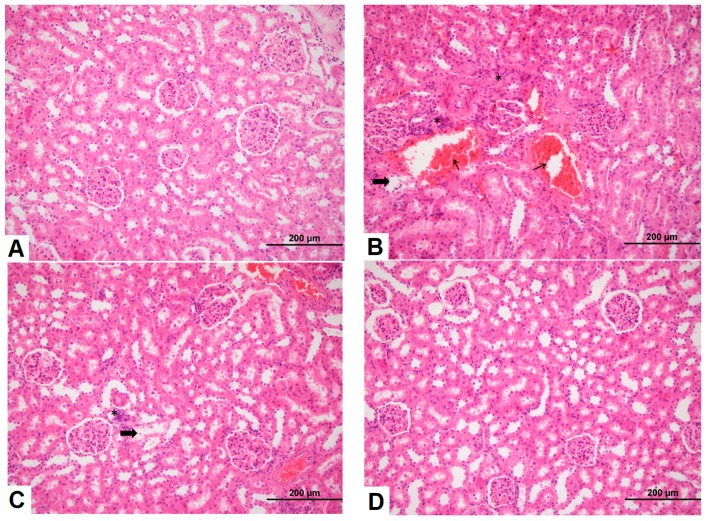
Pathological observations of kidney tissue in rats after modeling for 24 h (hematoxylin-eosin (H&E) staining, 200×). (**A**) Control group; (**B**) CIN group; (**C**) NAC group; (**D**) Sildenafil group (arrow: hemorrhage,*: mononuclear cell infiltration, thick arrow: tubular damage).

H&E staining of kidney tissues showed that the renal tubular epithelial cells of the control group presented normal morphology and structure, as shown in Figure 3. However, CIN markedly increased hemorrhage, shedding of the brush border, tubular vacuolization and degeneration, infiltration of mononuclear cells, and intratubular obstruction by granular casts, as detected in the CIN-treated rat kidneys compared to the control group. Specifically, the most severe alterations were observed in the renal cortico-medullary boundary zone. Moreover, renal injury in the sildenafil-treated CIN group had fewer histological changes than the NAC-treated CIN group.

**Figure 4:**
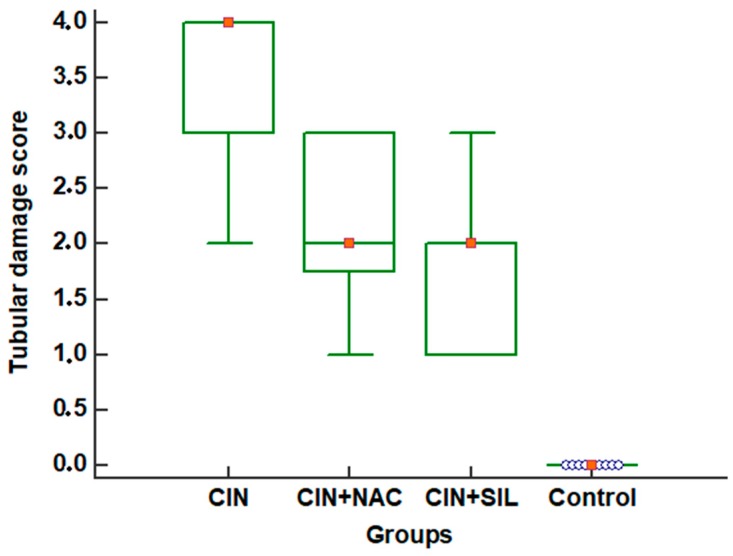
Tubular damage scores of rats after modeling for 48 h. According to multiple comparison tests (post-hoc test: Tukey), the difference between control group and other groups and CIN and CIN *+* NAC groups were significant (*p* < 0.05) (Table 2).

In Figure 4, comparative tubular damage scores for all groups are shown with box-plot graphics.

According to groups, tubular damage scores were significant (*p* < 0.001). According to the multiple comparison test, all possible dual comparisons were significant (*p* < 0.05) (Table 1). According to the same test, the differences between the control group and other groups, and CIN and CIN *+* NAC groups were significant (*p* < 0.05) (Table 2).

### 3.4. Observation of Renal Immunohistochemistry for Four Groups

**Figure 5:**
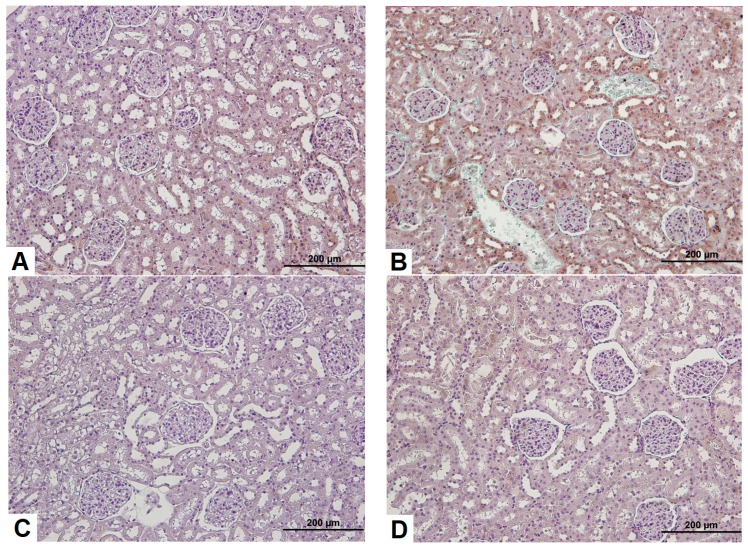
Immunohistochemistry of HIF2α in rat kidney section after modeling for 24 h (200×). (**A**) Control group; (**B**) Model group; (**C**) NAC group; (**D**) Sildenafil group.

The conventional immunohistochemistry method was used to perform HIF-2α immunohistochemical staining on paraffin sections. As observed under a light microscope, the tubules in the control group presented a very low immunoreactivity intensity for HIF-2α positive tubules, and the staining was lighter than the CIN group, as shown in Figure 5.

**Figure 6:**
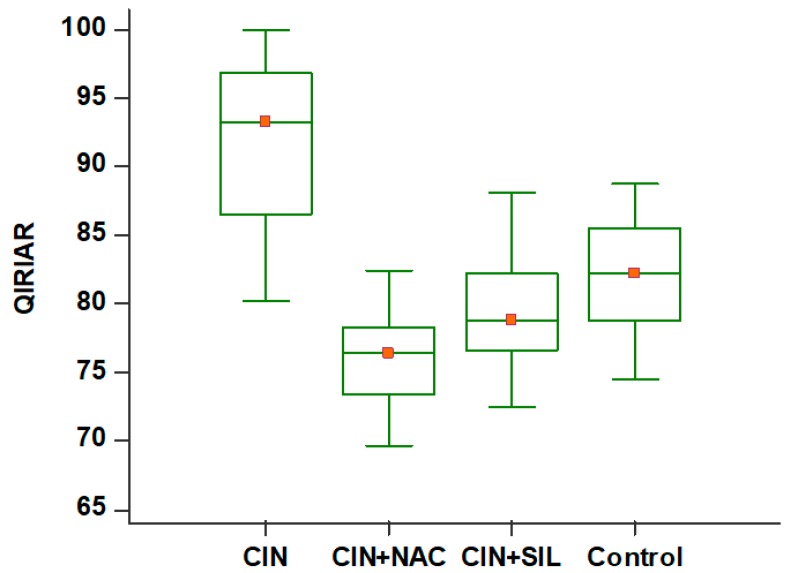
HIF-2α immunostaining in CIN model kidneys of control, CIN, and treated groups. Ratio of HIF-2α-positive tubular epithelial cells with immunolocalization staining in rats after modeling. According to the multiple comparison test (post-hoc test: Tukey), all possible dual comparisons were significant (*p* < 0.05). QIRIAR: Quantitative immunohistochemistry score.

In Figure 6, comparative QIRIAR counts are given for all groups and are shown with box-plot graphics.

## 4. Discussion

Owing to the increased use of iodine contrast agents worldwide, CIN is quickly increasing in frequency. Though it is prevalent in all age and patient groups, CIN risk is increasing among patients with diabetes and hypertension [1,21]. In the current literature, the rates of CIN were reported to be between 0.2–2.0% after contrast tomography was taken by emergency services [1]. Generally, along with increased creatinine levels for 3–5 days, it may be deleterious in the long-term, and dialysis requirements may arise. In the current study, in order to form a CIN model, a non-ionic low-osmolar contrast medium agent was used, as in the study of Sun et al. [17]. In all rats in the experiment model, CIN was developed significantly compared to the control group. The effects of HIF-2α in CIN and treatment groups were demonstrated by using the modified CIN protocol of Sun et al. [17], with biochemical parameters, histopathological analyses, and immunohistochemical tests.

For the biochemical parameters, serum BUN and Cr, and urine BUN and Cr levels were significantly increased in the CIN group; however, only serum Cr and urine BUN levels were significantly decreased in treatment groups. Similar to our results, Wang et al. [16] detected a decrement in increased serum Cr levels in the CIN group after treatment with prostanoids.

Globally, the primary aim of various studies was conducted to reveal the pathogenesis of CIN are related to diagnosis and treatment. In recent years, clinicians, especially those working in emergency services, have conducted studies in order to increase the awareness of CIN. Due to a number of difficulties in CIN diagnosis and treatment, BUN and Cr are the most commonly preferred biochemical markers for diagnosis. These markers are necessary for the diagnosis of CIN; however, they are not sufficient to demonstrate the efficacy of treatments or the extent of ischemic injury.

In the current study, HIF-2α was studied on rats modeled with CIN, and here it was used both as a diagnostic agent and also to evaluate treatment efficacy. Our study is among a number of preliminary studies to determine HIF-2α levels in rats modeled with CIN. A number of studies have been performed on a number of biomarkers related to CIN [1].

As the underlying mechanism for contrast agents to induce CIN is complex, diagnosis and treatment for CIN are also complex situations. In clinical treatments, two different mechanisms are mainly used. The first is the sufficient hydration of the patient, and the second is antioxidant treatment [1,22]. The most commonly used antioxidant treatment is NAC, which scavenges ROS and increases the vasodilatative effect of nitric oxide [2]. Despite the exact mechanism against renal damage induced by contrast agents being unknown, NAC is the most widely used agent in the world for CIN treatment, due to its renal protective effects and antioxidant properties [2]. Specificially, NAC may efficiently decrease oxidative stress formed with contrast agents [2]. In the current study, rats with CIN formation, HIF-2α is significantly increased in both tissue and serum. After treatment with NAC for 48 h, a significant decrease was detected in HIF-2α levels. NAC also may protect kidneys by more than one mechanism, such as removal of ROS, induction of glutathion (GSH) synthesis, and stabilization of nitric oxide [1]. Apart from NAC, many other antioxidants such as sildenafil and vitamin C are used for the treatment of CIN [21,23]. Thus, we used NAC to compare with sildenafil as a positive control drug. For performance comparisons, we measured both the treatment efficacies of NAC and sildenafil.

Sildenafil is a vasoactive agent used for erectile dysfunction and pulmonary artery hypertension in humans, besides being used in pig models during cardiac by-passes and in rat models for gentamicin-induced nephrotoxicity [23,24]. De Almeida et al. [15] regarded sildenafil to be successful in preventing nephropathy in CIN-formed rats. In the current study on CIN-modeled rats, sildenafil treatment efficiently protected renal function, decreased both serum and tissue HIF2 α levels, and also decreased sCr levels; however, it did not affect the BUN, urine BUN, or urine Cr levels. In the histopathological evaluation, we detected significant differences in rats modeled with CIN.

Important evidence obtained from experimental studies indicates that chronic hypoxic damage of tubulointerstitium is a common eventual pathway to induce the progression of chronic kidney disease to end-stage renal disease [2]. Thus, therapeutic intervention to prevent hypoxia may be a valid way to terminate the progression of CIN. HIF is an essential intermediate for defense mechanisms against hypoxia [25]. HIF-α accumulates in the cell and moves to the nucleus, and by binding to the *β*-subunit, undertakes functions in erythropoesis, angiogenesis, cell metabolism, cell growth, and apoptosis [6]. In chronic and acute renal failure, HIF is activated. There are studies referring to HIF activation to be responsible for renal fibrosis in chronic renal failure [6]. HIF is efficient in preserving the hypoxic tissues, decreasing hypoxia and decrementing the injury [9]. In hypoxic states, there is no oxygen available for molecular hydroxylation. In states such as CIN, there is acute renal damage that is secondary to hypoxia, such as a decrease in intramedullar blood flow, and direct tubular damage [9,17].

In general, oxidative stress was revealed as an important factor [2]. Thus, several antioxidant agents are important factors for the mechanism of oxidative stress [6]. Although an exact consensus does not exist, in practice, NAC is the most widely preferred agent [1]. After their injection into the body, contrast agents produce oxygen radicals through pathophysiologic effects. Contrast agents primarily cause vasoconstriction that plays a direct role in the production of oxygen radicals, adenosil residues, and calcium ions. Afterwards, the glomerular basal membrane and mesangial cells are damaged, and oxygen radicals are formed by the increment in leukocyte chemotaxis and xanthine oxidase activity. Oxygen radicals are claimed to be the causative factor for CIN due to contrast agents, and these molecules may lead to toxic ischemic reaction or tissue damage related to the immune system [1,22,23]. In the current study, for the model group, we detected significantly increased HIF-2α levels after modeling, and the levels were significant in renal tissue.

Our study revealed that the HIF activation in the CIN model and CIN treatment had histopathological and immunohistochemical effects. Several studies have demonstrated HIF-2α activation in renal ischemic models [7,11,26]. In the current study, the rat models with CIN and the rats treated with NAC and sildenafil, HIF-2α activation was measured, and differences were detected. In the CIN model, HIF-2α activation was determined, and in the CIN *+* NAC and the CIN *+* SIL models, this activation was decreased versus the CIN model. Kong et al. [27] demonstrated late-phase renal tubular HIF-2α activation to be protective for renal fibrosis and renal dysfunction, and also demonstrated its use as a therapeutic agent in the late phase of chronic kidney disease.

In the current study, HIF-2α levels measured in tissues were as follows: 49.11 ± 15.74 ng/mL in the control group, 71.082 ± 13.086 ng/mL in the CIN group, 44.881 ± 9.735 ng/mL in the CIN *+* SIL group, and 31.638 ± 6.448 ng/mL in the CIN *+* NAC group, respectively. Accordingly, the increase in HIF-2α levels in the CIN group versus the control group was significant. Zheng et al. [28], in ischemia/reperfusion injury mice model, detected increased HIF-2α levels in the kidneys. Again in the same study, treatment with sevoflurance induced a significant decrease in HIF-2α levels.

In our study, the decrease in HIF-2α levels in treatment (CIN *+* SIL, CIN *+* NAC) groups versus the CIN group was significant. HIF-2α levels measured in serum and also in tissue were significant between groups. Urine HIF-α levels were non-significant in treatment groups versus the control group. All these measured values may be used to evaluate the efficacy of treatment with HIF-2α levels in CIN treatment. Similarly, BUN levels were non-significant for CIN and treatment groups. Conversely, sCr levels were significant in treatment groups versus the CIN group. Urine BUN levels were significant for CIN *+* NAC and CIN groups; however, they were non-significant for the CIN *+* SIL group. There were no significant differences for urine Cr in treatment groups versus the CIN group. Our current results related to BUN and sCr were consistent with the current literature [1,18,23].

## 5. Conclusions

In the current study, HIF-2α levels were significantly increased in the CIN model. After CIN treatment with NAC and sildenafil, HIF-2α levels were significantly decreased. NAC and sildenafil efficiently reduced renal injury due to contrast agent implementation. Increased HIF-2α levels in CIN formation and decreased HIF-2α levels after treatment may be beneficial in monitoring and treatment of patients with CIN. The underlying mechanism for the change in HIF-2α levels states—where CIN or acute renal damage is presumed—may be associated with a decrement in regional reactive oxidative stress and renal pathological changes. Thus, these conclusions may be used to construct an experimental base for the use of HIF-2α levels in clinical prevention and treatment of CIN. Despite the use of NAC and sildenafil in CIN treatment, we determined NAC treatment to be more significant.

### Restrictions of the Study

There are few studies in the literature measuring HIF-2α levels after applying contrast materials, and this current study is one of the first studies of its kind that has been performed on rats.

Though suggestions for measuring the level of HIF-2α several times to indicate contrast nephropathy were made; due to budget restrictions, only one round of HIF-2α could be measured in this study.

## Figures and Tables

**Table 1:** Comparison of four groups according to laboratory variables.

Variable	Groups				*p*
	Control	CIN	CIN *+* SIL	CIN *+* NAC	
BUN (mg/dL)	21.440 ± 2.45	17.610 ± 1.61	22.889 ± 1.965	20.111 ± 1.90	<0.001
sCr (mg/dL)	0.380 ± 0.34	0.344 ± 0.03	0.294 ± 0.022	0.283 ± 0.025	<0.001
Urine Urea (mg/dL)	63.250 ± 122.94	170.000 ± 245.85	164.111 ± 202.093	135.333 ± 224.997	0.678
Urine Cr (mg/dL)	1.220 ± 2.43	2.000 ± 4.09	2.333 ± 3.000	1.000 ± 3.000	0.788
HIF-2α-tissue (ng/g)	49.110 ± 15.74	71.082 ± 13.086	44.811 ± 9.735	31.638 ± 6.448	<0.001
HIF-2α-serum (ng/mL)	5.770 ± 2.01	8.430 ± 1.330	5.252 ± 1.206	3.627 ± 0.839	<0.001
HIF-2α-urine (ng/mL)	0.024 ± 0.006	0.044 ± 0.453	0.025 ± 0.007	0.0441 ± 0.453	0.382
QIRIAR	82.159 ± 0.437	91.864 ± 0.634	76.076 ± 0.378	79.423 ± 0.366	<0.001

Results are expressed as mean ± SD. SIL: Sildenafil; CIN: contrast-induced nephropathy, NAC: *N*-acetyl cysteine, sCr: serum creatinine, mg: milligram. QIRIAR: Quantitative immunohistochemistry score.

**Table 2:** Multiple comparison of tubular damage scores of rats after modeling for 48 h.

Variable	Groups				*p*
	Control	CIN	CIN *+* SIL	CIN *+* NAC	
**Tubular damage score**	0 (0–0)	4 (2–4)	2 (1–3)	2 (1–3)	<0.001

Results are expressed as median (min-max).
